# Monitoring of sedation depth in intensive care unit by therapeutic drug monitoring? A prospective observation study of medical intensive care patients

**DOI:** 10.1186/s40560-018-0331-7

**Published:** 2018-09-14

**Authors:** Richard J. Nies, Carsten Müller, Roman Pfister, Philipp S. Binder, Nicole Nosseir, Felix S. Nettersheim, Kathrin Kuhr, Martin H. J. Wiesen, Matthias Kochanek, Guido Michels

**Affiliations:** 1 Department III of Internal Medicine, Heart Center, University Hospital of Cologne, Kerpener Str. 62, 50937 Cologne, Germany; 2 Center of Pharmacology, Department of Therapeutic Drug Monitoring, University Hospital of Cologne, Gleueler Str. 24, 50931 Cologne, Germany; 3 St. Katharinen-Hospital GmbH, Kapellenstrasse 1-5, 50226 Frechen, Germany; 4 Institute of Medical Statistics and Computational Biology, University of Cologne, Kerpener Str. 62, 50937 Cologne, Germany; 5 Department I of Internal Medicine, University Hospital of Cologne, Kerpener Str. 62, 50937 Cologne, Germany; 6 Department of Cardiology, University Hospital of Cologne, Kerpener Str. 62, 50937 Cologne, Germany

**Keywords:** Analgosedation, Intensive care, Richmond Agitation-Sedation Scale, Drug monitoring, Midazolam, Sufentanil

## Abstract

**Background:**

Analgosedation is a cornerstone therapy for mechanically ventilated patients in intensive care units (ICU). To avoid inadequate sedation and its complications, monitoring of analgosedation is of great importance. The aim of this study was to investigate whether monitoring of analgosedative drug concentrations (midazolam and sufentanil) might be beneficial to optimize analgosedation and whether drug serum concentrations correlate with the results of subjective (Richmond Agitation-Sedation Scale [RASS]/Ramsay Sedation Scale) and objective (bispectral (BIS) index) monitoring procedures.

**Methods:**

Forty-nine intubated, ventilated, and analgosedated critically ill patients treated in ICU were clinically evaluated concerning the depth of sedation using RASS Score, Ramsay Score, and BIS index twice a day. Serum concentrations of midazolam and sufentanil were determined in blood samples drawn at the same time. Clinical and laboratory data were statistically analyzed for correlations using the Spearman’s rank correlation coefficient rho (*ρ*).

**Results:**

Average age of the population was 57.8 ± 16.0 years, 61% of the patients were males. Most frequent causes for ICU treatments were sepsis (22%), pneumonia (22%), or a combination of both (25%). Serum concentrations of midazolam correlated weakly with RASS (*ρ* = − 0.467) and Ramsay Scores (*ρ* = 0.476). Serum concentrations of sufentanil correlated weakly with RASS (*ρ* = − 0.312) and Ramsay Scores (*ρ* = 0.295). Correlations between BIS index and serum concentrations of midazolam (*ρ* = − 0.252) and sufentanil (*ρ* = − 0.166) were low.

**Conclusion:**

Correlations between drug serum concentrations and clinical or neurophysiological monitoring procedures were weak. This might be due to intersubject variability, polypharmacy with drug-drug interactions, and complex metabolism, which can be altered in critically ill patients. Therapeutic drug monitoring is not beneficial to determine depth of sedation in ICU patients.

**Electronic supplementary material:**

The online version of this article (10.1186/s40560-018-0331-7) contains supplementary material, which is available to authorized users.

## Background

Critically ill patients on intensive care units require regimens of analgosedation for several reasons such as mechanical ventilation. Finding the optimal treatment and sedation depth is often challenging because of multimorbidity and polypharmacy. High interindividual variability concerning pharmacokinetics as well as insufficient monitoring can lead to inadequate dosage of drugs, which might increase morbidity and mortality. Low states of analgosedation can cause hypercatabolism, immunosuppression, hypercoagulopathy, awareness and increased sympathetic activity, or inadvertent extubation, whereas deep sedation can be responsible for extended mechanical ventilation, higher risk of nosocomial pneumonia, increasing costs, and neuropsychological dysfunction [1–6]. Hence, monitoring of analgosedation is an elementary part of ICU procedures to avoid excessive sedation states, drug-induced delirium, and higher mortality [2, 4, 6]. According to the guidelines, the current state of analgesia, sedation, and delirium should therefore be measured every 8 h using validated monitoring procedures [4].

To improve individual treatment, clinical scores such as the Richmond Agitation-Sedation Scale (RASS) Score [7] and Ramsay Sedation Scale Score [8] (Additional files 1 and 2) as well as neurophysiological monitoring procedures such as BIS monitoring have been established. BIS monitoring is based on simplified electroencephalograms (EEG) and a consecutive spectral analysis [9, 10].

Gold standard for the assessment of sedation depth is the RASS Score in combination with physiological parameters like heart rate, blood pressure, mimic, gesture, lacrimation, and perspiration [4, 6]. Reliability and validity of the RASS Score have been analyzed in several studies [7, 11]. Particularly in deeper sedated patients, RASS Score is more precise than Ramsay Score, which is not recommended in the German AWMF guidelines anymore [4].

The BIS index is a unitless value ranging from 0 to 100, a value of 100 representing an adequate awake condition (Additional file 3) [12]. Several authors have shown that BIS index has a good validity and reliability regarding the RASS and the Ramsay Scores [3, 13–15].

The combination of benzodiazepines and opioids is a common regime in European ICUs, although nonbenzodiazepine sedatives should be preferred [4, 6]. In comparison with other benzodiazepines, the advantages of midazolam are its rapid metabolic inactivation, clearance, and comparatively short elimination half-time [16]. If the prolonged intravenous application is expected, sufentanil is superior to fentanyl because of its additional hypnotic potency [6, 17–19]. Sufentanil has a strong affinity to μ_1_-receptors causing a potent analgesic effect. Compared to other opioids, affinity to μ_2_-receptors, which induces respiratory depression, is lower [20]. Hence, both drugs are suitable for ICU therapy.

The correlations between serum concentrations and subjective monitoring procedures (RASS and Ramsay Scores) as well as objective monitoring procedures (BIS-monitoring) were investigated in this study. The aim of this study was to clarify whether or not therapeutic drug monitoring is useful to assess the sedation depth in intensive care patients.

## Methods

### Patient population

This study was performed between December 2012 and December 2014 in cooperation with the ICU of the Department of Internal Medicine and the Center of Pharmacology, University Hospital of Cologne. Intubated, artificially ventilated, and analgosedated intensive care patients, who agreed to this study by themselves or through legal representatives before intubation, were included. Exclusion criteria were age < 18 years, missing patient’s consent, history of alcohol or drug abuse, history of neurological or psychiatric conditions, polytraumatization, conditions after CPR, and suspicion of hypoxic brain damage. RASS Score, Ramsay Score, BIS index, and serum concentrations of analgosedatives were measured twice a day (7:00 a.m. and 7:00 p.m.). Overall, 49 patients were included in the study, and 538 data points were determined. The maximal period under consideration was 10 days. Sepsis was defined according to the criteria by Bone et al. [21].

### Procedure of intubation and maintenance of analgosedation

After induction with fentanyl, etomidate and rocuronium orotracheal intubation was performed. Analgosedation was then maintained with midazolam (infusion rate of 0.03–0.2 mg*/*kg*/*h i.v.) and sufentanil (infusion rate of 0.1–1.0 μg*/*kg*/*h i.v.). According to the clinical presentation and sedation depth, the infusion rates were adapted.

### Assessment of depth of sedation

Sedation depth was evaluated by RASS Score, Ramsay Score, BIS monitoring, and measurements of serum concentrations of analgosedatives. To avoid artifacts, BIS index was recorded after 15 min of patients’ rest, and averaging time was set at a maximum of 30 s. Afterwards, RASS and Ramsay Scores were assessed. Finally, blood samples were taken. For calculation of RASS Score, initially, the decision had to be made whether a patient was “awake” (positive values) or “sedated” (negative values). “Awake” patients were assessed regarding the reaction while the observer was entering the room. If the patient was considered to be “sedated,” further evaluation was made using a fixed protocol in order to cause eye-opening or a change in facial expression: observer entering room, verbal contact, light physical contact, severe physical contact by shaking patient’s shoulder, induction of light pain by pinching the back of patient’s hand, and induction of severe pain by rubbing patient’s sternum.

### Measurement of the drug serum concentrations

A liquid chromatography-tandem mass spectrometry (LC-MS/MS) method for quantitative serum concentration measurements of four analgosedatives (ketamine, lorazepam, midazolam, and sufentanil) frequently used in intensive care medicine has been previously developed and validated according to ICH Guidelines Q2 (R1) [22]. This technique was successfully applied on adult and critically ill patients and provides the basis for pharmacokinetic research projects. The results of this test are available within 2 to 4 h.

### Statistics

Statistical analysis and graphic design were performed using IBM SPSS Statistics version 22. Correlations were analyzed using Spearman’s rank correlation coefficient rho (*ρ*). The value of *ρ* was interpreted as follows: 0 ≤ |*ρ*| < 0.1—no or very weak correlation; 0.1 ≤ |*ρ*| < 0.5—weak correlation; 0.5 ≤ |*ρ*| < 0.8—moderate correlation; 0.8 ≤ |*ρ*| ≤ 1—strong correlation. Box plots were used for graphic illustration.

## Results

### Patient population structure

Clinical data and baseline characteristics of the patient population are listed in Table 1. The average age of the study population was 57.8 ± 16.0 years. Sixty-one percent of the patients were males. About two thirds of the patients were suffering primarily from a hematooncologic condition. Indications for ICU treatments were manifold. Most frequent reasons were sepsis (22%), pneumonia (22%), or a combination of both (25%). Eight percent of the study population had no prior diseases and required ICU treatment due to an acute medical problem.

**Table 1:** Clinical data and baseline characteristics of the study population

Baseline data		
Total number of patients	*n* = 49	
Average age (years) ± SD (range)	57.8 ± 16.0	(20–83)
Gender	19 women/30 men	
Weight (kg) ± SD (range)	87.9 ± 27.7	(60–210)
APACHE II Score ± SD (range)	13.1 ± 6.7	(2–27)
SOFA Score ± SD (range)	17.8 ± 3.5	(9–23)
Endotracheal ventilation	*n* = 49	
Analgosedation with sufentanil and midazolam	*n* = 49	
Total number of blood samples	*n* = 538	
Number of blood samples per patient (range)	11.0	(3–20)
Underlying disease	*n*	%
Hematooncology	31	63.3
COPD	7	14.3
Nephrology	3	6.1
Infectiology	2	4.1
Cardiology	2	4.1
None	4	8.2
Reason for ICU treatment	*n*	%
Pneumonia	11	22.4
Sepsis	11	22.4
Sepsis + pneumonia	12	24.5
Sepsis + acute renal failure	2	4.1
ARDS	2	4.1
GvHD	1	2.0
Acute renal failure	3	6.1
Cardial decompensation	2	4.1
Hb-relevant bleeding	1	2.0
Pneumonia + acute pancreatitis	1	2.0
Mesenterial ischemia	1	2.0
Coecum perforation	1	2.0
Pneumonia + upper intestinal bleeding	1	2.0

### Correlations between subjective monitoring procedures, objective monitoring procedures, and serum concentrations of analgosedatives

**Fig. 1:**
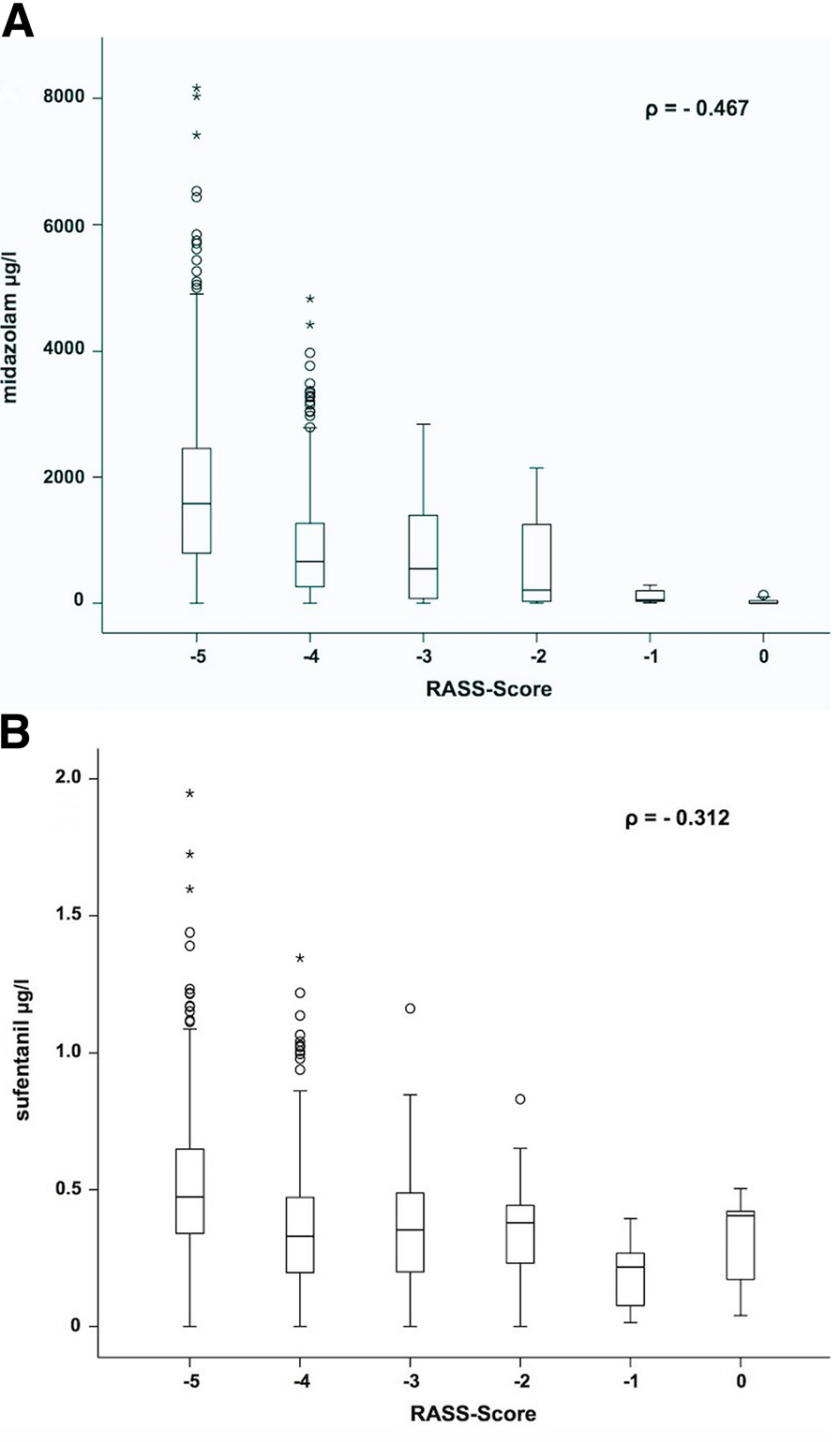
Depiction of the correlation between RASS Score and serum concentrations of midazolam (**a**). Depiction of the correlation between RASS Score and serum concentrations of sufentanil (**b**)

**Fig. 2:**
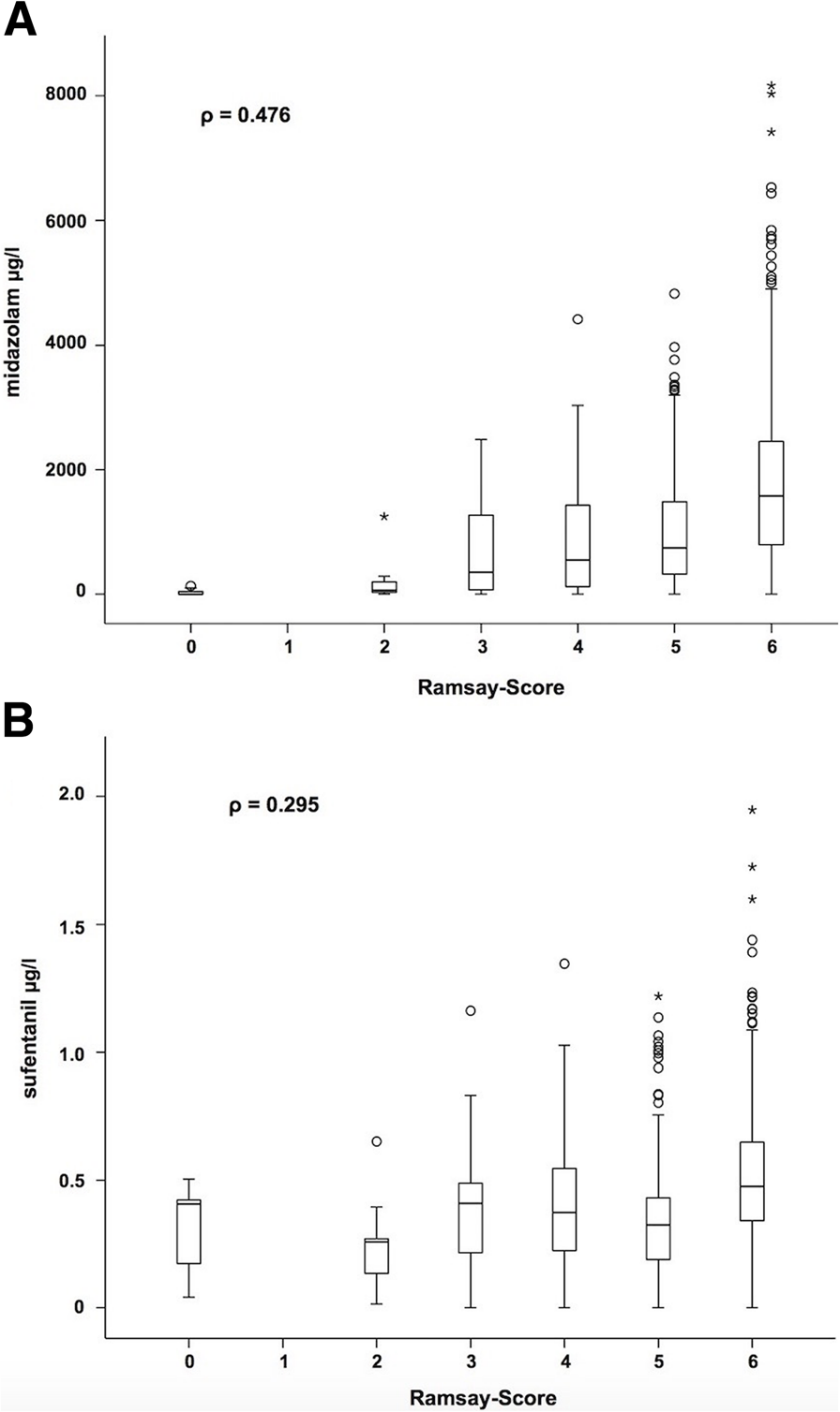
Depiction of the correlation between Ramsay Score and serum concentrations of midazolam (**a**). Depiction of the correlation between Ramsay Score and serum concentrations of sufentanil (**b**)

The correlation between RASS Score and serum concentrations of midazolam reached a *ρ* value of − 0.467 (Fig. 1a). A weak correlation was observed between RASS Score and serum concentrations of sufentanil (*ρ* = − 0.312, Fig. 1b). Higher serum concentrations of analgosedatives are tendentiously associated with lower RASS Scores. Similar results were observed concerning Ramsay Score, which correlates also only weakly with midazolam serum concentrations (*ρ* = 0.476, Fig. 2a) and sufentanil serum concentrations (*ρ* = 0.295, Fig. 2b). Overall correlations between subjective monitoring procedures and serum concentrations of the investigated analgosedatives were low.

**Fig. 3:**
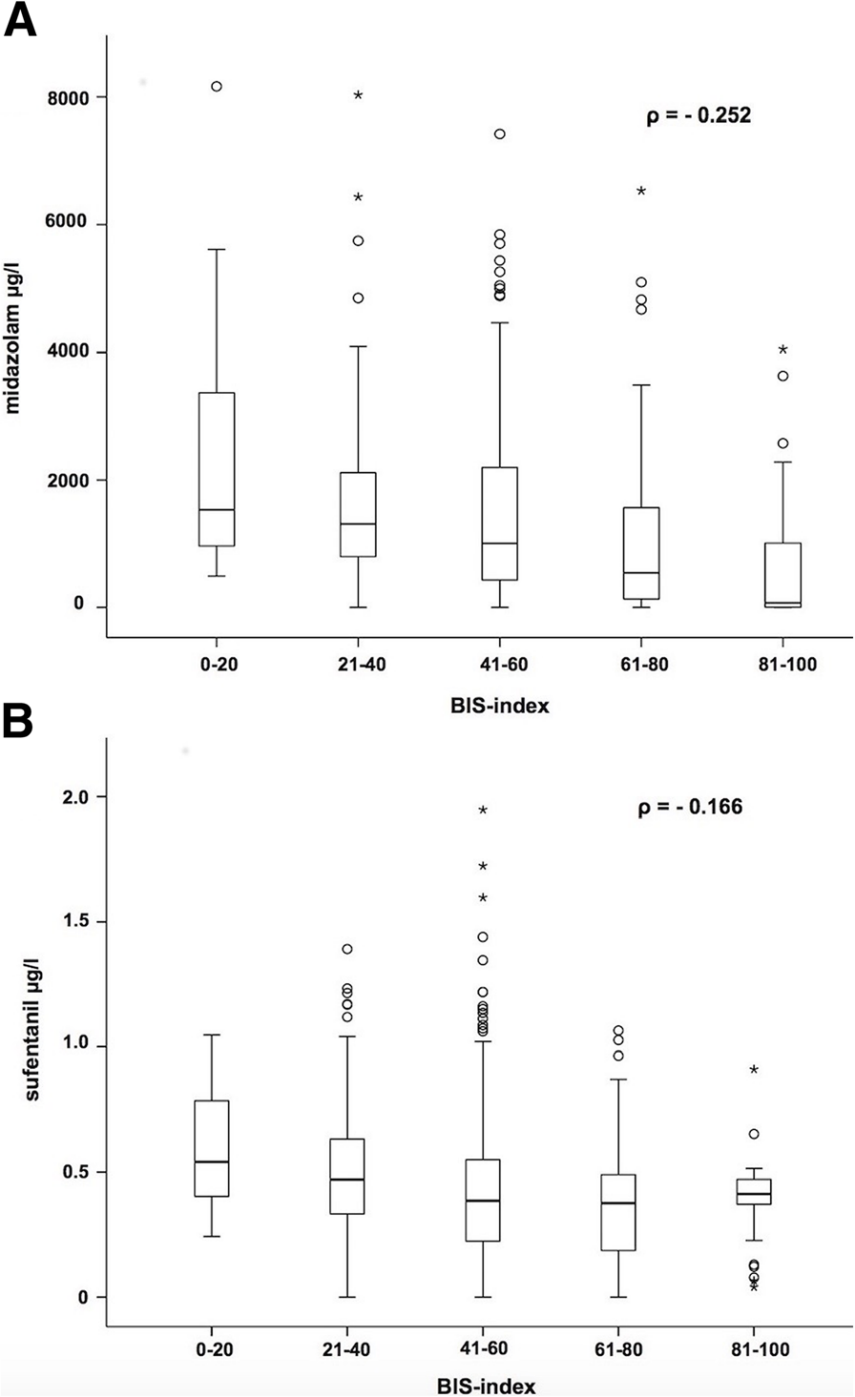
Depiction of the correlation between BIS index and serum concentrations of midazolam (**a**). Depiction of the correlation between BIS index and serum concentrations of sufentanil (**b**)

Correlations between BIS index and serum concentrations of midazolam (*ρ* = − 0.252, Fig. 3a) and sufentanil (*ρ* = − 0.166, Fig. 3b) were only weak. Nevertheless, higher blood levels of midazolam were observed with falling BIS index values.

## Discussion

Since light sedation levels are associated with improved clinical outcomes, monitoring procedures are part of the ongoing research. To avoid adverse clinical events due to excessively low or deep sedation, the purpose of this study was to analyze whether the measurement of drug serum concentrations might lead to a highly individual, drug concentration-guided analgosedation. Therefore, serum concentrations were compared to common monitoring procedures.

### Correlation between RASS/Ramsay Score and drug serum concentrations

Serum concentrations of sufentanil had only a weak correlation with RASS and Ramsay Scores, whereas serum concentrations of midazolam showed a better but still weak correlation. Bremer et al. [23] investigated 648 critically ill patients by therapeutic drug monitoring, who received a combination of fentanyl and midazolam when they had to be mechanically ventilated > 24 h. The authors found a strong correlation between midazolam plasma concentrations and sedation levels (*r*^2^ = 0.906). A Ramsay Score of 6 was observed in patients with a median midazolam level of 594 ng/ml, and high intersubject variability was seen. Similar results were described by Glass et al. using the Observers’ Assessment of Alertness/Sedation Scale (OAA/S Score; *r* = 0.746) [24].

In this study, serum midazolam levels correlated weakly with RASS and Ramsay Scores. A Ramsay Score of 6 was associated with a median midazolam concentration clearly above 1000 ng/ml, whereas the other patients showed median midazolam concentrations lower than that.

Bremer et al. [23] described a significant increase of midazolam plasma levels in critically ill patients within the first days due to reduced midazolam clearance mainly caused by impaired liver function. Park and Miller [25] found reduced cytochrome P450 3A4 (CYP3A4) activity in critically ill patients, which is a hepatic key enzyme for the midazolam pathway. Prolonged sedation additionally caused by an accumulation of conjugated 1-hydroxymidazolam was also observed in septic shock patients with severe renal failure [26, 27]. Bolon et al. [28] showed that in such cases, dialysis is rather effective in eliminating the conjugated metabolite than midazolam itself. Therefore, for patients needing dialysis, liver function plays a key role in midazolam clearance. Furthermore, Vinik et al. [29] observed a higher portion of unbound midazolam in patients with renal failure causing prolonged sedation, even when free drug clearance was unchanged.

Comedication with opioids could inhibit midazolam metabolism [30]. Moreover, the impact of age on the midazolam metabolism is commonly known, and the dosage has to be reduced in the elderly. However, surrogate parameters to guide the adaption of infusion rates such as serum bilirubin and serum creatinine levels rise with a delay of more than 10 days [23].

In this study, 57% of the patients suffered at least from sepsis or acute renal failure, which led to high intersubject variability (Figs. 1a and 2b) and a slight correlation.

Ethuin et al. [31] analyzed the pharmacokinetics of long-term sufentanil infusion for analgosedation with midazolam in ten ICU patients. The mean sufentanil serum concentration to reach a Ramsay Score of at least 3 was 0.86 ± 0.60 ng/ml. In this study, median serum concentrations of sufentanil were between 0.25 and 0.5 ng/ml (Fig. 2b) independent of the sedation depth determined by Ramsay Score. Correlations between RASS/Ramsay Score and serum concentrations of sufentanil were weak. Since sufentanil is an analgesic drug with only a hypnotical side effect, it is not surprising that the correlations with clinical scales are lower than with midazolam, which is a primary sedative drug. In the study of Glass et al. [24], none of their patients lost consciousness (OAA/S Score, 0–2) receiving alfentanil solely. Because midazolam and sufentanil were given simultaneously, the analysis of the isolated clinical effect of each drug is limited and confounded. Wappler et al. [18] investigated the efficacy of a three-level regimen of analgosedation in patients during ICU treatment: sufentanil mono (short-stay, group 1), sufentanil + midazolam (long-term intubated patients, group 2), and sufentanil + midazolam + clonidin (group 3). Adequate sedation was defined by a Ramsay Score of 2–3, which was reached in all groups. However, sufentanil infusion rates were higher in groups 2 and 3, which showed that polypharmacy contributes to intersubject variability. Additionally, continuous drug infusion leads to longer elimination half-times of sufentanil compared with single bolus use caused by increased tissue distribution, changes in protein binding, and often impaired hepatic function in critically ill patients [31]. Hofbauer et al. [17] investigated sufentanil requirement of elderly patients undergoing ventilatory support in ICUs and concluded that no adjustements have to be made regarding the patients’ age.

### Correlations between BIS index and drug serum concentrations

Miyake et al. [32] investigated the correlation between serum concentration of midazolam and BIS index in 24 orthopedic patients (ASA I/II). Patients were separated in a small dose (0.2 mg kg^−1^) and a large dose midazolam group (0.3 mg kg^−1^). After remifentanil, midazolam, and vecuronium were administered, intubation was performed, and eight blood samples were collected within 1 h before the operation. Although midazolam plasma concentrations were significantly higher in the large dose group, the authors found no differences concerning BIS index between the two groups. This indicates that there is no correlation between BIS index and serum concentrations of midazolam. In this study, BIS index and midazolam serum concentrations showed only a weak correlation (*ρ* = − 0.252), which stands in line with the results of Miyake et al. [32]. A limitation of both studies is that concentrations of the active metabolite were not measured. Glass et al. [24] observed a decreasing BIS index at higher midazolam serum concentrations. Maximum midazolam serum concentration was around 800 ng/ml, whereas in this study, much higher concentrations were found (Fig. 3a). Concerning analgosedation, a BIS index between 55 and 70 seems to be adequate [14]. Several authors described that midazolam can only cause a decrease of BIS index to 65–70 [33, 34]. Miyake et al. [32] found a correlation between BIS index and the relative beta ratio in EEG, which indicates that BIS index is influenced by cerebral beta activity. Billard et al. [35] described that midazolam induces an increased EEG frequency and amplitude. Seven out of eight patients showed an increase in relative beta power in EEG. Bagchi et al. [34] detected a marked divergence between BIS index and a subjective monitoring evaluation (OAA/S Score) in sedation protocols with midazolam. Approximately 38% of their patients sedated with midazolam were deeply sedated based on OAA/S Score, whereas BIS index value remained at 70. The time to reach a BIS index of 70 was significantly longer in the midazolam group compared with a propofol group. Ibrahim et al. [33] also found that BIS index is a better predictor for sedation with propofol than with midazolam. However, in this study, BIS index below 70 occurred frequently (Fig. 3a), which might be explained by the combination of pharmacons. Ben-Shlomo et al. [36] showed that midazolam and opioids act as a supraadditve concerning sedation.

Conclusively, BIS index will reach its limits—especially as a primary monitoring of sedation depth—when the effect of midazolam is monitored because it does not further decrease although the patient is clinically sedated and plasma concentrations are higher than needed for adequate sedation.

Glass et al. [24] showed that BIS index correlated with hypnotic drug concentrations, whereas alfentanil at plasma concentrations < 300 ng/ml did not effect it. Despite increasing alfentanil serum concentrations (maximum, approximately 280 ng/ml), BIS index did not decrease and remained high. However, Billard et al. [35] showed that BIS index might be suppressed below 50 at higher doses of alfentanil. Guignard et al. [37] investigated how remifentanil levels influence BIS index in a pain-free steady state of propofol and during a painful intervention (orotracheal intubation). In all patients, BIS index remained stable before intubation, which means remifentanil did not influence BIS index. This might be explained by the fact that hypnotics have a higher impact on EEG than opioids, which unfold their effect through an inhibition of subcortical structures. Patients with lower remifentanil infusion rates showed an increase in heart rate, mean arterial pressure, and BIS index during intubation, which stand in line with the observations made by Iselin-Chaves et al. [38], who described an inverse correlation between BIS index variability and level of analgesia. In contrast to that, Kato et al. [39] calculated clearly a better correlation between the RASS Score and the BIS index when low-dose remifentanil was administered in addition to propofol. In this study, high variable BIS index values were observed at almost the same serum concentrations of sufentanil (Fig. 3b).

### Limitations of the study

This study was a single-center study with a relatively small population of 49 intubated patients. Moreover, BIS index values are very susceptible. For example, endotracheal or oral suctioning, body hygiene procedures, passive movements, and physical contact are able to influence BIS index without changing the sedation depth necessarily [10, 40]. To minimize this interference, BIS index was recorded after a period of patients’ rest. Nevertheless, the level of noise in ICUs is significant and cannot be completely avoided to ensure the patients’ safety. Pharmacokinetics of midazolam and sufentanil vary with disease severity such as sepsis with higher distribution volume and especially hypalbuminaemia due to capillary leaking. Septic shock patients often suffer from kidney and liver dysfunction, which lead to a dysregulated drug metabolism [27, 41]. CYP3A4 is a key enzyme for the midazolam and sufentanil metabolism. Its activity can be altered by CYP interactions caused by other drugs such as antibiotics, which were not monitored in this study. Further potential drug-drug interactions in ICUs are likely and often underestimated [42].

Delirium may influence the assessment of sedation. However, we did not screen our patients for delirium since Haenggi et al. [43] reported that even in patients with a RASS Score of − 2/− 3, delirium is overdiagnosed and difficult to be differentiated from sedation. Therefore, many factors contribute to an almost unpredictable interindividual variability of drug serum concentrations and its effects.

## Conclusion

Correlations between drug serum concentrations (midazolam and sufentanil) and RASS Score, Ramsay Score, or BIS index were only weak, the results for midazolam being slightly better than those for sufentanil. This might be due to the intersubject variability, polypharmacy with drug-drug interactions, and complex metabolism, which can be altered especially in critically ill patients. Therefore, individual course of disease and patients’ comorbidity have to be taken into account. Therapeutic drug monitoring is not beneficial to determine the depth of sedation in ICU patients. Analgosedation of patients in ICUs should therefore be guided by subjective monitoring procedures.

## Additional files

Additional file 1: Richmond Agitation Sedation Scale [7]. (PDF 50 kb)

Additional file 2: Ramsay Sedation Scale [8]. (PDF 44 kb)

Additional file 3: Monitoring the depth of sedation with BIS-monitoring [12]. (PDF 44 kb)

## Abbreviations

APACHE: Acute Physiology and Chronic Health Evaluation
ASA: American Society of Anesthesiologists
AWMF: Arbeitsgemeinschaft Wissenschaftlicher Medizinischer Fachgesellschaften
BIS: Bispectral index
CPR: Cardiopulmonary resuscitation
CYP3A4: Cytochrome P450 3A4
EEG: Electro encephalograms
IBM SPSS: International Business Machines Statistical Package for the Social Sciences
ICH: International Council for Harmonisation
ICU: Intensive care unit
LC-MS/MS: Liquid chromatography-tandem mass spectrometry
OAA/S: Observers’ Assessment of Alertness/Sedation
RASS: Richmond Agitation-Sedation Scale
SOFA: Sequential Organ Failure Assessment
