# Supplementary material for: Monitoring of sedation depth in intensive care unit by therapeutic drug monitoring? A prospective observation study of medical intensive care patients
Source: J Intensive Care. 2018 Sep 14;6:62. doi: 10.1186/s40560-018-0331-7 (PMC6137863; doi:10.1186/s40560-018-0331-7)
Supplement: Supplementary file 1 — Richmond Agitation Sedation Scale [7]. (PDF 50 kb) [file 40560_2018_331_MOESM1_ESM.pdf]

Score	Term	Description
+ 4	combative	overtly combative or violent; immediate danger to staff
+ 3	high agitation	pulls on/or removes tube(s) or catheter(s) or has aggressive behavior toward staff
+ 2	agitated	frequent nonpurposeful movement or patient-ventilator dyssynchrony
+ 1	restless	anxious or apprehensive, but movements not aggressive or vigorous
0	alert and calm	
- 1	drowsy	not fully alert, but has sustained (more than 10 seconds) awakening, with eye contact to voice
- 2	light sedation	briefly (less than 10 seconds) awake with eye contact to voice
- 3	moderate sedation	any movement (but no eye contact) to voice
- 4	deep sedation	no response to voice, but any movement to physical stimulation
- 5	unarousable	no response to voice or physical stimulation
