# Supplementary material for: Monitoring of sedation depth in intensive care unit by therapeutic drug monitoring? A prospective observation study of medical intensive care patients
Source: J Intensive Care. 2018 Sep 14;6:62. doi: 10.1186/s40560-018-0331-7 (PMC6137863; doi:10.1186/s40560-018-0331-7)
Supplement: Supplementary file 2 — Ramsay Sedation Scale [8]. (PDF 44 kb) [file 40560_2018_331_MOESM2_ESM.pdf]

| Score | Description |
|---|---|
| 1 | anxious and agitated or restless or both |
| 2 | cooperative, orientated, tranquil |
| 3 | responsive to commands only |
| 4 | brisk response to light glabellar tap or loud auditory stimulus |
| 5 | sluggish response to light glabellar tap or loud auditory stimulus |
| 6 | no response to light glabellar tap or loud auditory stimulus |
