# Supplementary material for: Monitoring of sedation depth in intensive care unit by therapeutic drug monitoring? A prospective observation study of medical intensive care patients
Source: J Intensive Care. 2018 Sep 14;6:62. doi: 10.1186/s40560-018-0331-7 (PMC6137863; doi:10.1186/s40560-018-0331-7)
Supplement: Supplementary file 3 — Monitoring the depth of sedation with BIS-monitoring [12]. (PDF 44 kb) [file 40560_2018_331_MOESM3_ESM.pdf]

BIS-index	Clinic
100	awake patient
80	light-moderate sedation
60	general anesthetic
40	deep hypnosis
20	burst suppression
0	isoelectrical EEG
